# Rehabilitation of Executive Functions in a Real-Life Setting: Goal Management Training Applied to a Person with Schizophrenia

**DOI:** 10.1155/2012/503023

**Published:** 2012-10-11

**Authors:** M.-N. Levaux, F. Larøi, M. Malmedier, I. Offerlin-Meyer, J.-M. Danion, M. Van der Linden

**Affiliations:** ^1^Cognitive Psychopathology Unit, Department of Psychology, Cognition and Behavior, University of Liège, 4000 Liège, Belgium; ^2^Psychiatry Service I, Inserm 666 Unit, 67091 Strasbourg, France; ^3^Cognitive Psychopathology and Neuropsychology Unit, University of Geneva, 1211 Geneva, Switzerland

## Abstract

The aim was to assess the efficacy of a modified version of Goal Management Training (GMT) in a person with schizophrenia who had difficulties in attaining the final goal for new and multitasking daily-life situations. GMT is designed to improve abilities in establishing goal-directed plans and carrying them out effectively. Beneficial effects of GMT were measured for several clinical questionnaires, laboratory tasks, and three real-life situations: meal preparation (trained, familiar); washing (nontrained, familiar); meeting preparation (nontrained, unfamiliar). The results revealed improvement in planning and on trained laboratory and meal preparation tasks and a generalization of GMT effects on nontrained laboratory and everyday tasks. Self-esteem also improved. Finally, a two-year followup indicated the durability of the beneficial effects.

## 1. Introduction

A number of cognitive rehabilitation programs have been developed in order to remediate executive impairments in people with schizophrenia. Some interventions are designed to train several cognitive domains including executive functions (e.g., [1, 2]), while others target specific executive processes, such as problem-solving (e.g., [3]), cognitive flexibility, working memory, and planning (e.g., [4]). Formats include paper-and-pencil and/or computerized tasks which involve decontextualized material (e.g., shapes, numbers, texts) or more attractive formats with an audiovisual interface that are more game-like. The beneficial effects of these programs on executive functions are evident based on several meta-analyses on cognitive rehabilitation in schizophrenia (for a review, see [5]). However, the transfer of gains to the real world is small to medium and is variable across the (few) cognitive rehabilitation studies that included measures of psychosocial functioning [6, 7].

Typically, due to their structured character, cognitive rehabilitation tasks are disconnected from the situations people with schizophrenia meet in real-life. Indeed, everyday situations can be open ended, multitasking and engage a wide range of executive processes that are critical for the management of goals: the definition and the organization of subtasks according to an optimal order, the monitoring of multiple subtasks in parallel, the suppression of distracters, the detection of errors and their correction, delayed intention, and so forth. Many of these executive capacities, which are necessary for behavioral adaptation in daily life, are, however, very rarely addressed in laboratory exercises, yet difficulties with these capacities have been well documented in people with schizophrenia. For instance, Kessler et al. [8] identified deficits in specific executive functions (cognitive flexibility, inhibition of previously performed actions and control over a plan of actions) to explain everyday action disturbances in people with schizophrenia. Impact of executive impairments that is in attentional flexibility (shifting between self-generated goals and task-related stimuli), resistance to interference, and action sequencing abilities, was emphasized for particular activities of daily life (ADL) assessed in a real or computerized setting, such as choosing a menu, grocery shopping, and cooking a meal [9–11]. Surprisingly, in spite of their prevalence, goal-directed behavior difficulties meet by people with schizophrenia in their ADLs have not yet been the subject of cognitive rehabilitation programs, which in addition could orient the application of learned cognitive strategies in a real setting. 

One technique from the field of rehabilitation for neurologically impaired people, Goal Management Training (GMT; [12, 13]), provides a direct focus on goal-directed behavior difficulties in daily life. GMT is based on Duncan's theory of “goal neglect” [14, 15], which addresses dysexecutive self-regulatory deficits (i.e., failure to carry out intentional and apparently well-memorized actions). The theory of goal neglect maintains that any activity requires a list of goals that are used to impose coherence and structure to behavior and to create a plan of actions, allowing one to achieve goals. During task execution, actual situation and stated goal are compared, and appropriate actions are then selected and activated in order to reduce discrepancy. Inhibition of actions not contributing to the achievement of a goal and selection of new actions when anterior actions fail to attain the goal (flexibility ability) are two other important aspects of the theory. According to Duncan [14], much of the disorganized behavior in people with frontal cortex dysfunction can be attributed to difficulties in elaborating and implementing such lists of goals. 

The GMT procedure encompasses training 5 steps, each emphasizing one important aspect of goal-directed behavior: (1) stop: orient awareness toward the actual state of the situation; (2) define: the goal of the task; (3) list: the task into sub-steps; (4) learn: the steps; (5) check: if the result of an action corresponds to the stated goal. In the case of a discrepancy, the 5 steps are repeated. Therefore, the strategy consists of taking pauses during a current task to “stop and think”, selecting and maintaining the goal in memory while the task is executed, subdividing the goal in more simple and controllable subgoals, setting priorities for their execution, and using mental imagery or check lists. Exercises are administered in order to train people to use the strategy as a basis to evaluate their proper performance in ADL, implement new actions, and continuously monitor their success. GMT is a top-down approach that focuses on training processes that can be applied across domains and that encompasses different factors such as attention, problem definition, problem-solving, encoding and retrieval strategies, and monitoring. Thus, GMT aims to promote generalization of the training on activities not specifically addressed in a given intervention. The effectiveness of GMT has been demonstrated on several occasions such as in people with neurological lesions and in healthy elderly adults [13, 16–18]. In that people with schizophrenia also manifest goal-directed behavior difficulties in their daily life, GMT could prove beneficial for this clinical population as well. 

Thus, the aim of this study was to assess the efficacy of Goal Management Training (with some modifications from the original GMT described in Levine et al. [16]) in a person with schizophrenia. A single-case methodology for the cognitive rehabilitation was used for several reasons. First, since executive processes underlying goal-directed behavior can be differently impaired from one person to another, and the consequences of the deterioration in ADLs can occur in various ways according to the person's interaction with his/her environment. Thus, a personalization of the intervention is essential in order to target the person's daily-life needs in relation with his/her cognitive profile (for an example, see [19]). In the present study, GMT was specifically chosen for a person who presented a cognitive and functional profile adapted to this type of intervention: he had executive problems in several everyday domestic tasks, which essentially consisted of difficulties in organization when faced with new and multitasking situations and in attaining the final goal; while at the same time, he presented efficient intellectual functioning, intact working memory processes (storage and processing load), and sufficient attentional capacities to cope with information. Finally, a single-case study was also favored because this methodology allows a detailed investigation of the person's (cognitive and functional) profile in order to isolate the specific components involved in the training. 

The beneficial effects of GMT were assessed in real-life situations, where performance was evaluated with the help of behavior-scoring scales that provide an analysis of the cognitive processes underlying errors and, consequently, the mechanisms underlying the training effects. Indeed, qualitative and quantitative assessment of ADL dysfunction in a real setting has proven to be a fruitful approach in order to emphasize how specific cognitive difficulties of persons with schizophrenia affect particular behavior in complex ADL [10, 11, 20]. Two daily-life activities that were particularly difficult for the person were selected. Meal preparation was targeted in the rehabilitation program, while washing constituted a measure of generalization of GMT effects on a nontrained familiar activity. A third task, meeting preparation, was also used in order to broaden the assessment of generalization effects to a nontrained, albeit unfamiliar activity. Three laboratory tasks, which simulated everyday situations, were also included. Finally, several questionnaires were administered in order to assess the effects of GMT on anxiety, self-esteem, and clinical symptoms. 

## 2. Case Report

G. O. is a single 39-year-old man. He had finished his 11th year of secondary school when his first clinical symptoms (loss of communication, anxiety) appeared at around the age of 19. A diagnosis of undifferentiated schizophrenia, according to DSM-IV criteria (American Psychiatric Association [21]), was made at the age of 20. He has had 4 hospitalizations (total duration: 18 months), the last one being approximately 3 years before the beginning of the present cognitive rehabilitation program. For many years, G. O. lived with his mother before joining a sheltered house 8 years ago. At the time of the study, he lived with four house mates. Socially, G. O. frequented an association for occupants of sheltered houses and participated in different activities (artistic and written expression, relaxation, and gardening). His medication consisted of an atypical (haloperidol: 1 × 20 mg) and a typical (risperidone: 1 × 50 mg every 15 days) antipsychotic, one antiparkinsonian drug (procyclidine: 2 × 5 mg), and one antidepressant (escitalopram: 1 × 10 mg). He was seen by his psychiatrist about twice a month. His clinical profile was dominated by high levels of anxiety. Informed consent was obtained from G. O. for his participation to the study.

### 2.1. Daily Functioning

In order to assess G. O.'s daily-life functioning, the PROFINTEG general questionnaire [22] was administered to both G. O. and his reference caregiver in the sheltered house. This questionnaire was developed to evaluate, in people with cognitive impairment, approximately 80 activities of daily living divided into 10 categories: food; medication; housework; shopping; gardening; telephone; travelling; management; sewing; leisure. Both qualitative (screening questions are given in order to determine whether an activity has to be examined in detail or not) and quantitative (for each activity, difficulty scores range from 0 “no difficulty” to 5 “maximal difficulty”, and severity scores range from 0 “not important” to 3 “very important”) data can be collected. 

Three types of difficulty scores are defined for each activity: (1) a difficulty score for initiation is noted when the person does not spontaneously engage in an activity; (2) an omission score indicates that an entire subactivity has not been carried out within a given activity; (3) a score for an erroneous or inaccurate execution of one or several steps signifies that a subactivity has been incorrectly or inappropriately carried out. These three scores, each scored 1, are cumulative depending on the variety of the difficulties encountered in a given activity. Two others scores (exclusive with the previous ones) represent the person's inability to carry out the activity: a perseverance score (scored 4) is noted when the person persists in carrying out the activity, although one or several of the above-mentioned problems prevent him/her from successfully completing the activity; a total lack of autonomy (scored 5) indicates that the person no longer carries out a given activity because of his/her cognitive deficits. Finally, a severity score for each activity reflects the person's motivation to continue to carry out the activity. 

### 2.2. Results from the PROFINTEG General Questionnaire

On the basis of the questionnaire administered to G. O., some daily-life activities revealed difficulties (see Table 1). Regarding meal preparation, he always made the same easy dishes (i.e., meat cooked in the frying pan, potatoes, and frozen vegetables). Thus, he needed help from the caregiver to carry out new recipes and had difficulties in remembering recipes. He sometimes forgot to put certain ingredients in dishes and to defrost the meat. In general, G. O. needed to be reminded that the housework needed to be done. Regarding washing clothes, he often mixed different colors together and often forgot to add washing powder. Shopping lists and the shopping itself were done together with the housemates and caregivers. Concerning management of appointments, he was worried about forgetting them. Financial and administrative management were carried out with help from an administrator. He also mentioned difficulties in concentrating during reading activity. Furthermore, G. O. expressed that he was very worried about what he had to do during the day, and these concerns prevented him from concentrating. He was also “too early satisfied” with the results when cooking and doing the housework, which caused frequent remarks from caregivers. As a result, G. O. was anxious and lacked self-confidence regarding domestic tasks. He wished to carry them out more correctly and was motivated to remediate his difficulties.

According to comments made by the reference caregiver, G. O. was afraid of carrying out unfamiliar tasks and lacked flexibility in new situations. He rarely took initiatives and never carried out a new activity on his own. Moreover, he had a tendency to repeat the same questions several times a day. Nevertheless, he was capable of organizing himself when carrying out very common tasks. Regarding meal preparation, G. O. needed to be urged to do it. The meal was often ready before typical meal times and he forgot certain things, such as washing the vegetables. In general, G. O. needed to be reminded to do the housework, carried it out in a superficial manner or did not finish it, and could make errors such as mix different colored clothes together when washing clothes or added too much washing powder. He was very passive when writing up shopping lists and doing the shopping. Finally, he was easily distracted when watching TV.

In sum, difficulties of goal-directed behavior (lack of initiation, execution errors and omissions) were emphasized by both G. O. and his caregiver, and this concerned several domestic tasks including meal preparation, washing, and cleaning. 

### 2.3. Prerehabilitation Cognitive Assessment

A battery was administered to G. O., which covered various aspects of cognitive functioning. A score indicating a deficit was set at <−1.65 for Z-scores and at <10 for percentile scores (see Table 2). Performance was preserved on working memory tests assessing processing load and storage. Analysis of executive functions showed deficits related to flexibility, inhibition, and planning. Impaired performances were observed on auditory-verbal episodic memory tests, while performance on the visual nonverbal episodic memory test was intact. Attentional functions were impaired (phasic alert, divided attention, selective attention). G. O. did not show any difficulties in language flexibility. Nonverbal information processing speed was slow but not impaired. In sum, prerehabilitation cognitive assessment revealed impairments in flexibility, inhibition, planning, auditory-verbal episodic memory, and attentional functions. 

## 3. Rehabilitation Program

The goal of the cognitive rehabilitation program was established according to G. O.'s needs and objectives and consisted of working on one of the domestic chores which he had particular difficulties with, namely, preparing a meal. The execution of this task involves the ability to deal with executive functions, prospective memory, working memory, and multitasking, even for familiar tasks [23]. Based on the analysis of the results from different sources (cognitive assessment, PROFINTEG questionnaire, assessment of meal preparation, see below), G. O.'s difficulties when preparing a meal could be interpreted in terms of: a lack of flexibility and organization in complex or new situations (i.e., define a goal, sequence a plan of actions); problems in managing several things simultaneously; context neglect; a poor ability to maintain the pursuit of subgoals, to monitor their success and to attain the final goal; hyperactivity. In order to meet the rehabilitation objective, GMT seemed particularly appropriate for several reasons. First, this intervention was especially suitable for G. O. who presented satisfactory intellectual functioning, intact working memory processes (storage and processing load), sufficient attentional capacities, partial awareness of his daily-life difficulties, and a high level of motivation to remediate them. Second, components that were impaired in G. O. and that were related to his daily-life activities could be directly targeted with GMT. Third, the strategy could be implemented in daily-life situations, allowing an increase in G. O.'s self-determination (i.e., his intrinsic motivation and his sense of autonomy). Finally, the GMT strategy could be extended to various other organization-related ADLs difficulties that G. O. had. 

### 3.1. Procedure

The cognitive rehabilitation program lasted 16 sessions and was administered twice a week during an 8-week period. Each session lasted approximately 1.5 h and took place in G. O.'s sheltered house. The procedure was similar to the version described in Levine et al. [16] except that a psychoeducation session was added, there were more training sessions, and the content of sessions (described below) did not follow the script contained in the trainer's manual. Three stages were defined: (1) psychoeducation and learning GM steps; (2) training GM principles on pencil-and-paper tasks; (3) training in practical, real-life situations. 

The first session consisted of several objectives: (1) psychoeducation about executive functioning and the mechanisms underlying problems related to executive functioning in daily life; (2) an explanation of the aim and the theory underlying the GMT procedure; (3) an explanation of the steps of GMT. A sheet containing all this information was given to G. O. so that he could consult it when required. The second session was devoted to a discussion about the difficulties observed during the assessment of the meal preparation task in a real setting (see description below). The aim was to increase G. O.'s awareness about his difficulties and to explain, in a concrete manner, how applying GM could be helpful to him. During the third session, the 5 GM steps were learnt by using errorless learning and spaced retrieval [24]. Errorless learning has been proven to be effective in facilitating information processing stages in people with schizophrenia (e.g., [25, 26]). 

The aim of sessions 4 to 6 was to learn the implementation of the 5 GM steps on everyday paper-and-pencil tasks (see description below). For each exercise, G. O. was asked to fill out the first three GM steps on a sheet. Thus, he learnt to clearly define the situation, the goal to be achieved, and to list and order the substeps required to achieve the goal. He was then invited to carry out exercises with the help of the sheet and to regularly check progress as part of the mental checking routine. Concretely, after each completed substep, G. O. learnt to cross it out on the sheet and then to check his action. The sheet was used in order to provide G. O. with as much written support as possible as he presented difficulties in memorizing information. Finally, feedback on the performance was given. The aim of this part of the rehabilitation was to familiarize and promote an active application of the GM strategy on tasks that are less demanding and less complex than certain daily-life activities, such as meal preparation. 

During sessions 7 to 11, 14, and 15, G. O. learnt the application of the GM steps for the meal preparation task, which included different recipes graded in difficulty (from simple dishes, such as a hot salad, to a meal with a dessert). Finally, sessions 12, 13, and 16 were devoted to the implementation of the GM steps on domestic chores (cleaning the staircases and the living room). For each exercise regarding the meal preparation or domestic chores, G. O. was also asked to complete the first three GM steps on a sheet (to begin with, this was done during the session and afterwards and also as homework assignments before each session). The importance of estimating and managing the time was also emphasized and G. O. learnt to incorporate the time into the planning process. He then carried out the substeps with the help of the sheet and learned to systematically cross out and check each completed action. Furthermore, several homework exercises (i.e., statements describing a daily-life situation involving planning, such as going to the pharmacy to buy his medications, preparing his luggage to go on holiday) were also given to G. O. in order to teach him how to complete the GM steps himself, diversify the daily-life activities where GM could be applied, and allow him to become aware of the application of GM in a variety of situations. Homework was discussed at the beginning of each session. 

### 3.2. Design

The efficacy of GMT was measured with an A-B protocol consisting of 3 steps: a baseline phase (3 weeks); a rehabilitation phase (8 weeks); a postrehabilitation phase (3 weeks). Cognitive assessment was administered at pre- and postrehabilitation. The efficacy and specificity of GMT were assessed with different types of outcome measures: everyday paper-and-pencil tasks, everyday activities assessed in a real setting, and a control task. For each everyday paper-and-pencil and control task, six parallel measures were constructed (3 administered at baseline and 3 at postrehabilitation with a one-week interval between each measure) in order to minimize practice effects. The impact that GMT had on daily life was examined with several questionnaires (at pre- and postrehabilitation) assessing: anxiety, self-esteem, and clinical symptoms. 

## 4. Outcome Measures 

### 4.1. Everyday Paper-and-Pencil Tasks

In order to measure the efficacy of GMT, three paper-and-pencil tasks were administered that involved keeping goals in mind, analyzing the subgoals, and monitoring them (i.e., simulation of demand in real situations). These tasks were similar to those used in Levine et al.'s [16] study except that the time allowed to study the instructions was not limited. Two of these tasks (proofreading and room layout) were introduced in the rehabilitation sessions as examples of the application of GM principles.

**Proofreading**

G. O. received a text (a paragraph) and a list of three simple proofreading instructions on a separate sheet. The instructions involved underlining, circling, and crossing out words that met certain criteria (e.g., circle all numbers).

**Grouping**

G. O. received a sheet with two columns, each listing the age and sex (e.g., “25 M”) of 23 different individuals. Three instructions for grouping these individuals, based on age and sex, were listed on a separate sheet (e.g., place a check next to the females).

For both tasks, the instructions were removed from view after G. O. had memorized them, and the exercise sheet was given. G. O. was told to follow the instructions as quickly and as accurately as possible. The different measures for both tasks were the time spent in reading the instructions, the time spent in completing the task, the number of omissions, confusion errors (regarding instructions), and correct responses.

**Room Layout**

a 5 × 5 grid representing columns and rows (numbered from 1 to 5) of a seating scheme for a meeting was given to G. O. In each of the 25 cells, a letter (“A” to “E”) indicated an employee from one of five companies (company A to company E). The grid could be used to answer a series of five questions of ascending difficulty about the relative positions of company employees (e.g., what company is just above the “B” in Row “2”?). Measures were the time spent in answering the questions and the number of correct responses.

### 4.2. Meal Preparation Task

In order to evaluate the efficacy of GMT on meal preparation, an assessment in a real setting was carried out. This consisted of preparing a complete meal as quickly as possible based on a recipe that only described the main steps for each dish. Different recipes of equal difficulty between the pre- and postassessments were administered and were composed of: a meat, salad, vegetables, and a dessert. The task took place in the kitchen of the sheltered house during typical meal times. Instructions (see Appendix) were given to G. O. (orally and in written form) by the examiners who made sure that he had understood and had memorized them and, if necessary, they were explained to him again. G. O. could refer to a sheet with the instructions at any time. Two examiners were present in the kitchen in order to make a note of all the sequences of behaviors (according to a continuous observation schedule) and any comments or questions. The examiners only intervened when there was a potentially dangerous situation or if G. O. was completely stuck in an action. 

#### 4.2.1. Scoring System and Variables

G. O.'s behavior was quantified by using a decomposition scale (issued from the scales constituting the second part of the PROFINTEG instrument) constructed on the basis of a sequence of actions in order to optimally reach the goal of the meal preparation task (i.e., complete all the dishes as quickly as possible). This sequence of actions was subdivided into macrostructures (separate dishes that had to be prepared successively, that is, dessert, vegetables, meat, and finally the salad), which, in turn, were subdivided into microsteps (sequence of actions within the preparation of each dish). In order to detect errors, the sequence of G. O.'s actions was contrasted with the optimal sequence. Violations of the optimal sequence regarding macro- and micro-steps were considered. A cueing score was also noted for each subactivity. This score is a gradual indicator—0 (autonomy), 1 (general cueing), 2 (specific cueing), 3 (total cueing), and 4 (total dependence)—of the person's requirement for assistance in a given subactivity (for a detailed description, see Anselme et al. [22]).

Classification of errors was used based on Chevignard et al. [23, 27]. Errors were first classified on a descriptive level as 5 types of errors: omissions (any action or sequence of actions necessary to reach the goal omitted or incompletely performed), additions (any action or sequence of actions unnecessary for the completion of the task), inversion-substitutions (any action performed that is not part of the appropriate temporal sequence, or any object that is misused or inappropriate to the subgoal), estimation errors (poor estimation of the quantity, size, space, or time), and comments questions. Then, errors were classified according to the cognitive mechanisms underlying the occurrence of each error: control errors (inefficient monitoring of action), context neglect (failure to respect the instructions or the environment), environmental adherence (inappropriate action induced by the presence of an object), purposeless actions and displacements (a behavioral sequence not contributing to goal achievement), and dependency (any question relating to how to perform an action) (for a detailed description of type of errors, see Chevignard et al. [27]). The classification of errors was carried out independently by the two examiners, and the rating discrepancies were resolved through discussion. A qualitative analysis on the course of the activity and on the errors was also carried out. 

### 4.3. Washing Task

A washing task in a real setting was also included in order to measure if the effects of GMT transferred to another daily-life activities that G. O. also had difficulties with but which was not directly remediated. In order to minimize practice effects, washing white and light colored clothes was performed at prerehabilitation, whilst postrehabilitation consisted of washing dark colored clothes. For the pre- and postrehabilitation measures, the washing task included the same number of units to be washed at 30°C (i.e., clothes of the same color) and distracters (i.e., household linen to be washed at 60°C, clothes of a different color, etc.). The task took place in the laundry of the sheltered house, in the presence of two examiners. Instructions (see Appendix) were given to G. O. orally and in written form. A sheet with advice (e.g., how to choose the correct color of clothes, the ideal temperature, etc.) was also available in order to assess G. O.'s ability to interact with the environment. 

G. O.'s behavior was quantified by using a decomposition scale from the PROFINTEG instrument, which was based on an optimal sequence of actions to reach the goal of the washing activity (i.e., light and white (prerehabilitation)/dark (postrehabilitation) washing at 30°C). A cueing score was noted for each subactivity. Moreover, Chevignard et al.'s [27] classification of errors was used. A qualitative analysis on the course of the activity and on the errors was also carried out. 

### 4.4. Meeting Preparation Task

In order to broaden the assessment of the generalization of GMT effects to a task unfamiliar to G. O., a meeting preparation task was also evaluated in a real setting. G. O. had to prepare a meeting where 9 persons (a manager, a secretary, and 7 guests) would be present. He had to dispose the seating places (with name cards), drinks, and material (glasses, cups, notebooks, pens, laptop, microphone, projector, projection screen), while respecting the drink list (e.g., “Jean-Pierre wants orange juice and coffee”) and a set of 6 rules (e.g., “the manager is always placed at the front of the table with his/her laptop”, etc.). Distractor items were also included that are not necessary for the required task (e.g., other drinks, too many glasses or cups, name cards for guests that were not present). Moreover, a clock and a mobile telephone were necessary for the completion of the task (see below). For the pre- and postrehabilitation assessments, different rules of the same difficulty and different drinks and material (of the same quantity) were used in order to minimize practice effects. In addition to involving planning capacities, this task entailed prospective memory (i.e., get coffee from the kitchen 10 minutes after the beginning of the task) and updating in goal pursuit (i.e., remove the name card, material, and drink of the guest who withdrew from the meeting). This updating necessitated reorganizing places to take into account the rule “a woman (pre-rehabilitation)/man (post-rehabilitation) cannot be placed beside another woman/man”. These two components were controlled in the following manner: during the execution of the task, as soon as G. O. placed the name cards on the table, the phone rang announcing that the microphone would be used (prerehabilitation) or not (postrehabilitation) and that a guest decided not to attend the meeting. 

The task was carried out in a room in the sheltered house, in the presence of two examiners. Instructions (see Appendix) were given to G. O. orally and in written form. Three sheets with the instructions, the list of the persons and their drinks, and the rules, respectively, were given to G. O., and he was told that he could refer to them if needed. G. O.'s behavior was quantified with a decomposition scale similar to the one used in the PROFINTEG instrument and was based on an optimal sequence of actions to reach the goal of the activity (i.e., preparing the meeting while at the same time respecting the rules and the list of the persons and drinks). No cueing score was used for this task. Chevignard et al.'s [27] classification of errors was applied. A qualitative analysis on the course of the activity and on the errors was also carried out. 

### 4.5. Control Task

In order to measure if the rehabilitation program had a specific effect, and not a general training effect, the divided attention task from the Test for Attentional Performance (TAP; [28]) was administered at pre- and postrehabilitation. This task assesses the ability to share attentional resources simultaneously between two (visual and auditory) information-processing modalities and consists of four parallel versions to reduce test-retest effects. This task was chosen as a control task as G. O.'s performance on this task was impaired and since the program did not directly target processes underlying performance on this task.

### 4.6. Daily-Life Questionnaires

Anxiety was evaluated three times at pre- and postrehabilitation before each naturalistic assessment with the *State-Trait Anxiety Inventory *(STAI; French adaptation, [29]). The *Self-Esteem Inventory* (SEI; French adaptation, [30]) was administered to G. O. to assess his self-esteem. Finally, clinical symptoms were assessed using the *Positive and Negative Syndrome Scale* (PANSS; French adaptation, [31]) by an independent and experienced clinician. 

## 5. Results

### 5.1. Application of GMT

In the first exercises carried on the paper-and-pencil tasks, G. O. showed great difficulties in answering questions relative to the first 3 GM steps (stop, define, and list). During the execution of the tasks, he also had difficulty in following and respecting the substeps and was impulsive. However, he was able to integrate the 5 GM steps with respective key questions into memory. Progressively, the exercises allowed G. O. to become aware of the advantages of the GM steps, in particular, the checking step that resulted in fewer errors. Throughout the exercises, he seemed more capable of managing the completion of the substeps. 

In the first sessions of the meal preparation task, G. O. presented difficulties in completing the second and third GM steps. Moreover, when the meal consisted of several dishes, he showed difficulties in prioritizing the substeps to determine the correct order (macrostructure). During the execution of the meal, it was necessary to recall the actions “crossing out and checking” in the beginning, but afterwards G. O. recalled them spontaneously. Some difficulties (e.g., omissions, multitasking) were emphasized in order for G. O. to become aware of the significance of applying the GM steps. Throughout the subsequent sessions, G. O. progressively listed more steps alone and made fewer errors. For the last session of the meal preparation task (which involved several dishes), G. O. successfully carried out the three GM steps and the substeps with only two omissions, and the order of dishes was correct. 

For the application of the GM steps with housework, the same difficulties were observed at the beginning: difficulties in precisely defining the goal, listing the substeps and systematically crossing out and checking each action. However, G. O. progressively completed more substeps and spontaneously checked his actions. Moreover, he expressed a feeling of doubt regarding whether or not the staircases or living room was sufficiently clean. A discussion followed which concerned the importance of following the listed steps to the end, and of systematically crossing out and checking actions in order to reduce levels of doubt. 

Finally, regarding homework, after a while G. O. correctly completed the first three GM steps alone. The definition of the goal sometimes lacked precision. The list of the substeps was progressively more detailed. The organization of the actions according to an optimal order sometimes necessitated readjusting. Moreover, G. O. perceived that the GM varied according to each new situation. 

### 5.2. Postrehabilitation Cognitive Assessment

The post-rehabilitation assessment (see Table 2) revealed improvements in planning and dominant verbal response inhibition (Hayling task). Impaired performance in flexibility, inappropriate response inhibition (go/no-go), and attentional functions (phasic alert, divided and selective) showed no change. Performance in working memory (storage and processing load) and language flexibility remained preserved. Finally, nonverbal processing speed remained slowed.

### 5.3. Daily Functioning

Regarding the pre- versus postrehabilitation comparison of quantitative indices on the PROFINTEG questionnaire (see Table 1), the number of difficult activities decreased by 10 (from 21 to 11), and the difficulty score decrease by 16 points (from 46 to 30). The decrease of difficulties concerned initiation, omissions, and execution. According to G. O.'s responses to the questionnaire, he felt capable of preparing a new meal or dessert with a minimum of help in applying the GM. He did not forget certain ingredients anymore, nor did he forget to defrost the meat. Regarding housework, G. O. was still afraid of not correctly performing these chores; however, he did not forget doing them. After the rehabilitation program, he did not forget to put washing powder in the washing machine. However, he continued to mix different colored clothes. The shopping list and the shopping continued to be done together with fellow housemates and his caregivers. He was no longer afraid of forgetting his appointments. Financial and administrative management were still dealt with by the administrator. Finally, difficulties in concentrating during reading activity were still present. 

Comparisons of pre- and postrehabilitation indices on the PROFINTEG questionnaire administered to G. O.'s caregiver (see Table 1) indicated that the number of difficult activities decreased by 2 (from 17 to 15) and the difficulty score decreased by 6 points (from 43 to 37). The decrease of difficulties concerned initiation and execution. Based on the caregiver's comments, G. O. did not seem to have any problems with meal preparation. Regarding housework, he did not seem to systematically apply the GM and still mixed colors when washing. For the shopping activity, G. O. seemed more active when preparing the shopping lists and during shopping. He took the initiative himself to change his appointments. Finally, he was still very distracted when watching TV. 

### 5.4. Everyday Paper-and-Pencil Tasks

#### 5.4.1. Stability of Outcome Measures

**Prerehabilitation Measures**

G. O.'s performances on the proofreading task were not stable across the three assessments except for the omissions score between the first and third baselines. Regarding the grouping task, all of G. O.'s performances were stable. Performances on the room layout task were not stable except for the omissions score between the first and second baselines and for the confusions score between the first and third baselines.

**Postrehabilitation Measures**

G. O.'s performances on the proofreading task were not stable across the three assessments except for the omissions score between the first and second baselines and for the confusions score between the second and third baselines. Regarding the grouping task, G. O.'s performances were all stable. Finally, performances on the room layout task were not stable except for the omissions scores (see Table 3).

#### 5.4.2. Outcome Measure Comparisons

Scores were averaged in order to obtain a valid and reliable representation of performances on the outcome measures at the two assessment times (pre- and postrehabilitation) (see Table 3). A statistical analysis using chi-square tests (for an example of the use of chi-square tests in single-case studies of cognitive rehabilitation, see [32]) was carried out to compare the scores (omissions, confusions errors, and correct responses) at pre- and postrehabilitation. 

At postrehabilitation, G. O. spent more time reading and integrating the instructions, as well as when completing the proofreading and grouping tasks. For the proofreading task, omissions decreased significantly (*χ* ^2^(1) = 14.17; *P* < .001). On the grouping task, there was a significant improvement for correct responses (*χ* ^2^(1) = 24.72; *P* < .001) and a significant decrease for omissions (*χ* ^2^(1) = 23.46; *P* < .001). No change was observed on the room-layout task. Moreover, G. O. mentioned that he tried to apply certain rules learnt during the rehabilitation, such as checking, but did not use them in a constant manner. 

### 5.5. Meal Preparation Task

At prerehabilitation, G. O. had no action plan before starting. The expected order of the dishes was not respected resulting in two macrostructure errors. During the completion of the recipe, G. O. tended to be very dependent on the examiners and required cueing on a number of occasions. He committed many context neglects (omissions, inversions, and estimation errors) and purposeless actions. At the end of the activity, G. O. was very tired and felt that the recipe was difficult, as he had to manage several things simultaneously. 

At postrehabilitation, before starting, G. O. correctly completed the first two GM steps. For the third step, he began to list the first substeps and then stopped because he indicated that he was lost and in a hurry to start the recipe. G. O. committed one macrostructure error. During the application of the recipe, G. O. followed and verified the substeps but sometimes forgot to cross them out. He was largely less dependent on the examiners and his actions necessitated less cueing. He commented on actions that he was doing or that he had to carry out (e.g., “preparation of the stewed apples lasts 30 minutes”). He took the details of the recipe more into account and consulted the recipe less frequently, which reduced the number of context neglect errors. He controlled the cooking time for the cake with his watch. However, purposeless actions increased, and a majority of these can be attributed to excessive checking of the cake cooking in the oven. At the end of the exercise, G. O. felt that he managed well without any help. Quantitative results at pre- and postrehabilitation are presented in Table 4.

### 5.6. Washing Task

At prerehabilitation, G. O. correctly put some of the white clothes (at 30°) into the machine but forgot to add other clothes (i.e., light colored clothes) even though this was included in the instructions. After that, he continued to make neglect context errors, as he did not take the advice sheet sufficiently into account. On 5 occasions, G. O. erroneously considered that the task was finished, resulting in numerous omissions. 

At postrehabilitation, G. O. did not complete the GM steps on a sheet due to difficulties in putting his ideas in writing. He committed fewer omissions as he consulted the instructions (this was not the case at prerehabilitation). He commented on his actions (e.g., “this shirt is cotton”). He no longer asked the examiners any questions during the task. However, it was necessary for the examiners to cue G. O. on two occasions when he prematurely ended the task. He also committed some inversion errors, which could be due to the fact that he read the advice sheet in a superficial manner. Quantitative results at pre- and postrehabilitation are presented in Table 4.

### 5.7. Meeting Preparation Task

At prerehabilitation, G. O. seemed hesitant about how to begin the task. He started by putting a glass of orange juice and then notebooks, pens, and pencils. He then placed the name cards (one extra) and thereafter the other drinks. However, he was not able to take all the rules into account and made mistakes when distributing the drinks. Moreover, after the phone call, he correctly removed the microphone and the name card of the guest who withdrew but not the drink. G. O. also performed some purposeless actions and went to get the coffee three minutes too early. Finally, he did not check his actions and seemed in a hurry to finish the task. Thus, G. O. did not sufficiently take into account the instructions and the rules resulting in numerous context neglect errors (omissions, inversions). Following questions about the task by the examiners, G. O. said that he had difficulties completing it and mentioned that he did not have an action plan before starting. 

At postrehabilitation, G. O. correctly completed the first two GM steps. He then read the instructions and the list of the persons and anticipated to look at his watch before starting. He then proceeded in a structured manner: he more frequently consulted the instructions and rules and less frequently consulted the list. He started to place the name cards while taking into account the placing rules. He then dealt with the drinks of each person according to the list and correctly placed the objects according to the rules. However, he omitted two objects (a sheet of paper and a pen). Finally, he verified if he had respected all the rules. During the task, he commented on the actions that he was doing or that he had to carry out and mentioned the rules that he had to respect (e.g., “I have to get the coffee at 11 h 30”, “I cannot place a man next to another man”). G. O. became aware that he had to get the coffee two minutes too late. Following the phone call, he correctly left the microphone on the table and removed the name card, drink, and material of the guest who withdrew from the meeting. However, he forgot to reorganize in order to take into account the rule that “a man cannot be placed beside another man.” At the end of the exercise, G. O. mentioned that he did not have an action plan before starting the task except to simply read the instructions, the rules, and the drink list. Quantitative results at pre- and postrehabilitation are presented in Table 4.

### 5.8. Control Task

G. O. was more rapid at postrehabilitation but his attentional performance was more variable. The number of errors decreased, but an increase in omissions was observed (see Table 5).

### 5.9. Questionnaires

A statistical analysis using chi-square tests was carried out to compare the scores on the questionnaires at pre- and postrehabilitation (see Table 6).

**Anxiety**

No significant change was observed on the state and trait scales of the STAI.

**Self-Esteem**

G. O.'s responses on the SEI indicated a significant increase for the total score (*χ* ^2^(1) = 9.78; *P* = .002).

**Psychiatric Symptomatology**

no significant change was found for the total score, positive and negative symptoms, and general psychopathology on the PANSS.

## 6. Followup 

A follow-up assessment took place two years after the end of the cognitive rehabilitation program in order to assess if the principles of GMT promoted the persistence of beneficial effects in G. O.'s daily-life functioning. During this period, G. O. remained clinically stable without any hospitalizations. As previously, he continued to see his psychiatrist twice a month, and his treatment was the same. Moreover, he did not take part in any kind of rehabilitation (i.e., cognitive rehabilitation, cognitive-behavioral therapy) during this follow-up period. 

The assessment consisted of the PROFINTEG instrument (general questionnaire) and a questionnaire about the use and the repercussions of the GMT strategy in G. O.'s daily life. They were administered to both G. O. and his reference caregiver in the sheltered house. 

Regarding the postrehabilitation versus follow-up comparison of quantitative indices on the PROFINTEG questionnaire (see Table 1), the number of difficult activities remained stable (from 11 to 10), and the difficulty score decreased by 8 points (from 30 to 22). The decrease of difficulties concerned initiation and omissions. According to G. O.'s responses to the questionnaire, he prepared a meal without errors and help, and he varied the recipes that he prepared. G. O. carried out housework with less difficulty but he still doubted how well it was done. He explained that he did not mix different colored clothes when washing. The shopping list and shopping were still done together with fellow housemates, but he was able to do some shopping alone. He managed his appointments himself; however, financial and administrative management was still dealt with by the administrator. Finally, difficulties in concentrating during reading activity were still present. Furthermore, G. O. felt more conscious of his everyday functioning profile and more self-confident in the realization of everyday activities. He did not remember all of the 5 GMT steps but explained that he paid more attention to his environment and verified his actions (but not systematically).

Comparisons of pre- and postrehabilitation indices on the PROFINTEG questionnaire administered to G. O.'s caregiver (see Table 1) indicated that the number of difficult activities remained stable (from 15 to 14) and the difficulty score decreased by 11 points (from 37 to 26). The decrease of difficulties concerned initiation and omission. Based on the caregiver's comments, G. O. did not have any problems with meal preparation. Regarding housework, he continued to make a few errors but did not mix colors when washing. Regarding shopping, G. O. was active when preparing the shopping lists and sometimes did his shopping alone. He managed his appointments by himself. Finally, he was still very distracted when watching TV. In a general manner, the caregiver estimated that G. O. structured his days better, improved himself in his capacity to observe his environment (e.g., wash the cooking area when it is dirty) and to organize it (e.g., he organized himself before hand washing the dishes). Moreover, he was more flexible in his behavior. Clearly, thus, his well-being had improved and he felt more self-confident. Finally, this progress continued to evolve. 

## 7. Discussion

The aim of this study was to assess the efficacy of Goal Management Training on the organization of ADL in a person with schizophrenia who had difficulties with these activities. During the rehabilitation program, G. O. showed clear progress in his ability to apply the GM in an autonomous manner. Results from the cognitive assessment revealed improvements in planning and verbal automatic response inhibition. The efficacy of the intervention was also demonstrated based on specific gains obtained on the trained laboratory proofreading task and the trained meal preparation activity. Furthermore, generalization of the GMT effects was observed on both the nontrained laboratory (grouping) and nontrained everyday (meeting preparation) tasks. Finally, G. O. reported an improvement in self-esteem following the rehabilitation. 

Cognitive assessment showed that GMT had a beneficial effect on two planning tasks and an inhibition task. On the Tower of London (TOL), a task that assesses the capacity to analyze and elaborate possible solutions to a new problem, a decrease of the subsequent execution time, and the number of the moves to reach the solution were observed. These results could be explained by a more fully formed plan, which resulted in improved accuracy. On the 6 Elements Test, G. O. had to attempt to do 6 simple subtasks within a limited time period, while at the same time respecting a number of rules. The goal was to apply an effective strategy, that is, engage in time management throughout the 6 subtasks and initiate task switching in order to complete the first items. After the rehabilitation, G. O. was able to organize the rules in order to perform all the subtasks efficiently. Finally, on the Hayling test, which assesses the ability to inhibit a strongly cued automatic response and to find an unrelated one, the number of errors greatly decreased. Before the rehabilitation, the observed deficit of response suppression could be due to the persisting use of routine semantic schema and a decrease in strategy use [33]. This observation could also be related to G. O.'s poor performance on the TOL at prerehabilitation where he showed an inability to inhibit an inappropriate move that was very strongly triggered by the context [34]. Thus, these results suggest that GMT improved G. O.'s ability to inhibit schemas induced by the context. Finally, the score on the errand test was impaired after rehabilitation. This may be due to the fact that, for this task, it was too difficult for G. O. to keep all the instructions in mind (i.e., make a plan and identify alternatives approaches).

Regarding the everyday paper-and-pencil tasks, proofreading and grouping, improved performance on these tasks were also associated with GMT. Indeed, G. O. read the instructions and performed the tasks more slowly and made fewer omissions. These results could indicate that GMT increased G. O.'s care and attention to the tasks, which in turn reduced errors. These results replicate those observed in Levine et al. [16], furthermore suggesting the specificity of the effects of GMT on these tasks. The beneficial effect was observed on the task (proofreading) that was incorporated into training, as well as on the task (grouping) that was not specifically addressed by the intervention. This result indicates that there was a generalization of the training effects to the laboratory tasks. On the contrary, no change was observed on the room layout task that was incorporated into training. Here, G. O. expressed difficulties in listing the substeps in order to formulate a plan. 

Beneficial effects of GMT were also demonstrated qualitatively and quantitatively on two ADL assessed in a real setting: meal preparation and meeting preparation. A considerable decrease of errors (omission, addition, and inversionsubstitution) was observed, and G. O. was less dependent on the examiners. Moreover, the analysis of errors provided evidence of the different mechanisms underlying the GMT effects. First, the reduction of context neglect errors and the more frequent consultation of the instructions and rules indicated that G. O. took the contextual information more into account. This could indicate that there was an improvement in his ability to analyze and deal with the environment, the first step of problem resolution [23]. This step has been compensated by GM strategy, which taught G. O. to direct his attention toward pertinent information and inhibit inappropriate responses. A less redundant consultation of the recipe in the meal preparation task and of the guest list in the meeting preparation task might also suggest that G. O. was able to better structure his interaction with information. Additionally, this context information processing enabled G. O. to revise an initial plan in the face of the external contingencies. For instance, in the meeting preparation task, G. O. removed the name card and material of the guest who withdrew from the meeting. Second, G. O. demonstrated that he was able to monitor ongoing actions, for instance in the meal preparation task where he controlled the cooking time of cake. Third, G. O. often used the checking step of GM strategy, allowing him to reduce the number of errors. This was particularly clear during the meeting preparation task, where he checked and monitored his actions very actively by reading the rules and by orally reciting them. Finally, he spontaneously made commentaries about actions that he was doing or that he had to do, which could be a sign of organization and verbal self-regulation [35, 36]. 

On the whole, these findings suggest specific effects of GMT on targeted processes: context information processing, ongoing response monitoring, checking, and verbal self-regulation. It is unlikely that these effects are due to mere practice on the tasks without having learned a specific strategy [13]. Moreover, on the control task that involved divided attention, the pattern of performance remained impaired indicating no general effect of GMT. The absence of improvement on different cognitive functions (flexibility, and attentional functions) that were impaired at prerehabilitation also demonstrates the specificity of GMT in our study. 

Generalization of GMT effects to other real-life contexts was manifested as improvement was observed on the meeting preparation task, a task that was not targeted during the cognitive rehabilitation program. Several issues can account for this effect. GMT is conceived as a top-down strategy, which can engage different behaviors and be applied to various contexts that need plan formulation. Moreover, generalization of learnt strategies was built into the intervention. Indeed, G. O. learnt how to apply GMT in contexts other than the target activity (i.e., meal preparation), such as domestic chores and different situations as illustrated in homework exercises. Finally, psychoeducation given before the training was very important in order for G. O. to become aware of the cognitive mechanisms involved in various ADL. 

G. O.'s self-esteem also improved after the intervention. For instance, before the rehabilitation, G. O. expressed lack of self-confidence about the completion of ADL. The implementation of the GM strategy in his daily-life enabled him to directly improve his self-confidence. On the contrary, results regarding clinical symptoms (the latter were not severe to begin with) did not show any significant improvement after the rehabilitation.

One key factor in the success of GMT was no doubt related to G. O.'s high level of motivation throughout the rehabilitation program (based on comments made by G. O. and the fact that he attended all the sessions). Motivation and rehabilitation engagement were in part promoted through the personalization of the rehabilitation goal and material, training of ADL, and verbal encouragement from the therapist. Indeed, according to Medalia et al. [5, 37], intrinsic motivation (desire to engage in an activity because it is inherently interesting and engaging) is an essential factor to consider as in a learning environment it could be associated with greater learning, higher self-esteem and a sense of well-being, and greater engagement. 

Some negative results following the application of GMT on the ADL deserve further comment. First, the beneficial effects of the GMT did not generalize to the washing activity. This might be related to the fact that this task was familiar to G. O. Indeed, he washed clothes once a week and he was used to doing it without sorting out according to temperature and color of clothes. This routine action relied thus on schema that specified in detail how the behavior should be carried out. In order to improve or change the behavior, G. O. had to reject the existing schema and create a new schema [23]. Limited impact of GMT on the washing task could be explained by G. O.'s difficulty in inhibiting the routine schema. It might be more difficult to spontaneously transfer training effects to routine actions, in contrast to new and nonroutine actions (such as the meeting preparation task), as new skills can be more easily and quickly incorporated [38]. Second, goal definition and splitting up of a task in substeps remained difficult steps to carry out by himself. In particular, G. O. had difficulty in mentally simulating the real execution of an action plan, which could be related to an observed defective ability in action sequencing, that is, detecting boundaries in large action units, such as macrosteps, and setting priorities among the events with regard to the stated goal. These difficulties could indicate a disturbance in causal connections between the component actions to represent a plan as a coherent and structured sequence of goal-related events [39, 40]. Consequently, the increased amount of fragmentized actions could have overloaded G. O.'s working memory capacity during the execution and monitoring of actions in ADL. GMT aimed to remediate these difficulties by teaching G. O. to define the main goal and to sequence the main goal into substeps by selecting the most appropriate steps for achieving the goal. G. O. also learnt to estimate and manage the time for the articulation of action plans. Moreover, when the steps of an activity were defined on a sheet of paper, G. O. could be guided by their automatic execution and monitoring (by following and crossing out each completed step on a sheet), decreasing the demands on working memory, and the intervention of voluntary control. Third, purposeless actions increased on the meal preparation task indicating that hyperactivity was still present in G. O. This could be related to his high level of anxiety that did not change after the rehabilitation and was predominant in his symptomatology. Finally, the caregiver's responses on the PROFINTEG questionnaire regarding G. O.'s daily functioning did not indicate a substantial decrease in difficulties, unlike G. O.'s responses. It should be noted that it was difficult for the caregiver to perceive everyday changes, as he only had brief contacts with G. O. mainly due to time constraints. 

Regarding the two-year followup, the durability of the beneficial effects was largely evident in that the beneficial effects of the cognitive rehabilitation program were still present. Indeed, G. O. still had fewer difficulties in the realization of everyday activities at followup. Additionally, G. O. expressed being more self-confident and autonomous—crucial goals of a rehabilitation program. However, it is important to note that a few rehabilitation sessions aimed at refreshing acquisition would have been necessary in order to favor a better internalization of the 5 GMT steps.

Several limitations can be mentioned. First, blind assessments were not carried out. However, this was not feasible as G. O.'s assessments were carried out as part of a cognitive rehabilitation program. We considered filming G. O. while performing the various tasks, but he did not approve of this. Nevertheless, all assessments were always conducted by two judges. Second, there was a lack of stability of performances on the proofreading and room layout tasks at pre- and postrehabilitation. 

Beneficial results from the different types of measures support evidence of the efficacy of GMT to structure goal-directed behavior in a person with schizophrenia. They suggest that GMT is a promising technique for the rehabilitation of everyday executive difficulties in this population. Future directions could be to analyze the effects of individual GMT steps with multiple baseline protocols (i.e., assessment after each step) and to use of a multiple case study design in order to determine the cognitive and functional profiles of those persons with schizophrenia who are susceptible (or not) to benefit from GMT (in its entirety or for specific steps). 

## Figures and Tables

**Table 1:** Indices and scores for the PROFINTEG general questionnaire.

Indices and scores	G. O.				Caregiver			
	Pre	Post	Diff.	Foll.	Pre	Post	Diff.	Foll.
Number of applicable activities (/87)	37	37	0	39	36	37	1	39
Number of difficult activities	21	11	−10	10	17	15	−2	14
Sum of difficulty scores	46	30	−16	22	43	37	−6	26
Activities with initiation difficulty	7	3	−4	1	9	7	−2	0
Activities with omission difficulty	8	4	−4	2	9	10	1	1
Activities with execution difficulty	6	3	−3	4	5	0	−5	10
Activities with perseverance	0	0	0	0	0	0	0	0
Activities with a lack of autonomy	4	4	0	3	4	4	0	3

Notes: Diff.: difference between post- and prerehabilitation; Foll.: followup.

**Table 2:** G. O.'s results on cognitive assessment.

Cognitive tests	Pre	Post
Working memory		
Digit span (forward) (MEM-III)	1.02	0.2
Digit span (backward)/number of trials for digit span (MEM-III)	2.4/0.67	0.59/0.33

Executive functions		
Inhibition:		
Go/no-go (TAP): median RT/SD RT/error(s)/omission(s)	**P1**/P27/P > 38/P > 4	**P7**/P14/P > 38/P > 4
Hayling: part A time/part B time/errors part B	**−3.39**/3.34/**−5.57**	1/1/2.91/1.51
Flexibility:		
Flexibility (TAP): median RT/SD RT/error(s)	**P1**/**P **<** 1**/P < 62	**P8**/**P1**/P < 82
Planning: Tower of London:		
N3: latency/total time/moves	−0.8/**−18.4**/−0.5	−0.8/**−1.9**/−0.1
N4: latency/total time/moves	0.1/**−2.7**/**−2.7**	1.3/−1/**−2**
I + 5: latency/total time/moves	0.5/0.5/0.9	0.7/1.1/0.9
N5: latency/total time/moves	−0.3/**−7.8**/−0.3	0.9/0.3/−0.5
I − 5: latency/total time/moves	−0.6/**−3.3**/**−3**	−0.6/−0.4/−1.3
N6: latency/total time/moves	0.2/−0.8/1.9	0.5/0.6/0.5
Six elements test: total score/error(s)	**−3.04**/**−1.7**	−0.19/0.72
Hotel test: tasks attempted/time allocation/time deviations	0.2/−0.16/1.01	0.2/0.19/1.01
Errand test: reference group	40%	**<10%**

Episodic memory		
Auditory verbal:		
Logical memory (MEM-III): (I) 1st recall/total recall/learning/theme	**3/−3**/1.67/**−2.67**	/
(II) Total recall/retention%/theme	**−3/−2/−1.67**	/
CVLT: 1st recall/5th recall/total recall/	−1.5/**P1-5/−2.74**	/
Short term recall/cued recall/delayed recall/delayed cued recall/	**P1-5/P1/P1-5/P1**	/
Recognition/recall B	P25/−1.13	/
Visual nonverbal:		
Face recognition (MEM-III): part I/part II/retention	0.33/1/0.67	/

Attentional functions		
Phasic alert:		
TAP: no signal: median RT/SD RT/signal: median RT/SD RT	**P4/P8/P2/P4**	**P5**/P54/**P7**/**P4**
Divided attention:		
TAP: median RT/SD RT/error(s)/omission(s)	**P4**/**P10**/**P4**/P > 79	P46/**P7**/P < 18/**P3**
Selective attention:		
D2: speed/accuracy/global performance/concentration	**P5**/P > 90/**P10**/P27	**P1**/P > 90/**P2**/P14

Processing speed:		
Digit Symbol-Coding (WAIS-III)	−1	−1.33

Language:		
verbal fluency: phonological/semantic	0.2/−0.64	0.22/0.38

Numbers in bold indicate a deficit score (<−1.65 for the *Z*-scores, *P* < 10 for the percentiles). RT: reaction time; SD: standard deviation.

Digit span, Logical Memory, Face recognition (MEM-III [41]); Go/no-go, flexibility, phasic alert; Divided attention (TAP; [28]); Hayling (French adaptation [42]); Tower of London (French adaptation [43]); Six Elements Test (French adaptation [44]); Hotel Test [45]; Errand Test [46]; California Verbal Learning Test (CVLT; French adaptation [47]); Digit Symbol-Coding (WAIS-III [48]); D2 [49]; verbal fluency [50].

**Table 3:** G. O.'s results on the everyday paper-and-pencil tasks.

	Prerehabilitation				Postrehabilitation			
	1	2	3	Mean	4	5	6	Mean
Proofreading								
Time to read instructions (sec.)	24	75	60	53	146	105	175	142
Time to complete task (sec.)	303	198	180	227	346	323	177	282
Omissions (/15)	6	0	6	4	2	1	0	1*
Confusions (/15)	0	1	4	1,67	9	0	0	3
Correct responses (/15)	9	14	5	9,33	4	14	15	11

Grouping								
Time to read instructions (sec.)	45	43	35	41	138	86	96	107
Time to complete task (sec.)	122	119	102	114	123	235	116	158
Omissions (/38)	9	9	6	8	0	0	0	0*
Confusions (/38)	0	0	1	0,33	0	0	0	0
Correct responses (/38)	29	29	31	29,7	38	38	38	38*

Room layout								
Time to answer (sec.)	197	206	170	191	187	177	173	179
Correct responses (/5)	5	4	2	3,67	3	5	4	4

*Significant effect at *P* < .01.

**Table 4:** G. O.'s results on the meal preparation, washing, and meeting preparation tasks.

	Meal preparation		Washing		Meeting preparation	
	Pre	Post	Pre	Post	Pre	Post
Total number of errors	44	28	14	7	19	5
Macrostep errors	2	1	/	/	/	/
Microstep errors	1	0	/	/	/	/
Omissions	10	3	7	4	5	3
Additions	10	14	1	0	4	0
Inversion substitutions	2	2	1	3	5	1
Estimation errors	5	1	0	0	0	0
Commentary questions	17	29	12	12	8	19
Control errors (CEs)	3	3	/	/	2	1
Context neglect (CN)	15	3	8	7	11	3
Environmental adherence (EA)	2	0	/	/	/	/
Purposeless actions (PA)	7	14	1	0	1	0
Dependency (D)	14	5	5	0	5	1
Duration of task (min.)	80	89	20	18	14	18
Time of latency before starting (sec.)	10	540	10	0	52	70
Number of actions	112	103	42	57	76	108
Frequency of recipe consultation	18	13	/	/	/	/
Frequency of instructions consultation	/	/	0	1	2	5
Frequency of advices/rules consultation	/	/	7	9	7	8
Frequency of list consultation	/	/	/	/	15	9
Cueing score	21	4	11	13	/	/
Score of dependency (cueing score + D)	35	9	16	13	/	/

**Table 5:** G. O.'s results on the control task (divided attention task).

	Prerehabilitation*				Postrehabilitation*			
	1	2	3	Mean	4	5	6	Mean
Reaction time median	799	777	784	786,67	630	736	614	660
	(P4)	(P4)	(P4)		(P46)	(P10)	(P54)	

Standard deviation	307,3	279,29	242,61	276,4	629,32	307,33	277,60	404,75
	(P10)	(P16)	(P27)		(P7)	(P10)	(P54)	

Errors	6	7	0	4,33	2	0	0	0,67
	(P4)	(P3)	(P > 73)		(P < 18)	(P > 73)	(P > 73)	

Omissions	0	3	2	1,67	5	4	4	4,33
	(<P79)	(P < 14)	(P < 24)		(P3)	(P7)	(P7)	

*Percentile scores in parenthesis.

**Table 6:** G. O.'s results on the subjective questionnaires.

Questionnaires	Prerehabilitation	Postrehabilitation
STAI		
State total (/80)	43	49
Trait total (/80)	51	51

IES		
Total (/50)	17	28*

PANSS		
Total (/210)	66	54
Positive symptoms (/49)	16	11
Negative symptoms (/49)	14	11
General psychopathology (/112)	36	32

*Significant effect at *P* < .001.

## Appendix

### A. Instructions for Everyday Tasks

#### A.1. Meal Preparation Task

“You will prepare a meal for yourself and your fellow housemates according to a recipe available on the kitchen table. All ingredients and utensils you need are in their usual places. On the table, you will also find the ingredients and utensils that you do not have at home. All the dishes must be ready at the same time, that is, with the least possible delay between the first and the last completed dish. We cannot help you. You must carry out the task as if you were alone.”

#### A.2. Washing Task

“You will wash a load of white and light colored (pre-rehabilitation)/dark colored (post-rehabilitation) clothes at 30°C. All the clothes are in the large wicker basket. When the washing program is finished, you must dry them in the tumble dryer. We cannot help you. You must carry out the task as if you were alone. A sheet of paper with advice on how to wash clothes according to color and temperature is available. Throughout the activity you can consult it as well as the sheet with instructions.”

#### A.3. Meeting Preparation Task

“You will prepare a meeting where a certain number of persons will be present. You have a list of rules at your disposal, as well as a list of the persons present and their respective drinks. All the necessary materiel and drinks are on a shelf, except the coffee that you must get from the kitchen 10 minutes after beginning the task. Regarding the use of a microphone, you will receive a phone call from the secretary to confirm, or not, its use during the meeting. Try to do the task as quickly and accurately as possible. We cannot help you. You must carry out the task as if you were alone.”
